# Natural additives and agricultural wastes in biopolymer formulations for food packaging

**DOI:** 10.3389/fchem.2014.00006

**Published:** 2014-02-26

**Authors:** Arantzazu Valdés, Ana Cristina Mellinas, Marina Ramos, María Carmen Garrigós, Alfonso Jiménez

**Affiliations:** Department of Analytical Chemistry, Nutrition and Food Sciences, University of AlicanteAlicante, Spain

**Keywords:** additives, active packaging, nano-biocomposites, legislation as topic, food wastes

## Abstract

The main directions in food packaging research are targeted toward improvements in food quality and food safety. For this purpose, food packaging providing longer product shelf-life, as well as the monitoring of safety and quality based upon international standards, is desirable. New active packaging strategies represent a key area of development in new multifunctional materials where the use of natural additives and/or agricultural wastes is getting increasing interest. The development of new materials, and particularly innovative biopolymer formulations, can help to address these requirements and also with other packaging functions such as: food protection and preservation, marketing and smart communication to consumers. The use of biocomposites for active food packaging is one of the most studied approaches in the last years on materials in contact with food. Applications of these innovative biocomposites could help to provide new food packaging materials with improved mechanical, barrier, antioxidant, and antimicrobial properties. From the food industry standpoint, concerns such as the safety and risk associated with these new additives, migration properties and possible human ingestion and regulations need to be considered. The latest innovations in the use of these innovative formulations to obtain biocomposites are reported in this review. Legislative issues related to the use of natural additives and agricultural wastes in food packaging systems are also discussed.

## Introduction

The food packaging industry is showing increasing interest in the development of new materials and processes to ensure that the food contained is safe and healthy and to improve shelf-life, which is an essential issue in the modern distribution and commercialization strategies. In addition, the use of commodities has greatly increased in the last decades in food packaging applications. Among the wide variety of materials currently used in food packaging, polymers have taken a major share because of their versatility and advantageous performance/cost ratio. However, most polymers used for food packaging are not biodegradable or compostable and therefore represent an increasingly serious end-of-life disposal problem worldwide. Although food package stability during the shelf-life of the product is an advantage, it turns into a disadvantage when the packages enter the post-use phase. In consequence, the use of polymeric materials obtained from non-renewable resources (which are usually non-biodegradable) in food packaging applications presents important environmental impact and waste generation issues. Packaging waste accounted for 71.6 million tons or 29.5% of the total municipal solid waste (MSW) in 2009 in the USA (http://www.epa.gov/osw/nonhaz/municipal/pubs/msw2009-fs.pdf). Accessed June 2011) and 56.3 million tons or 25% of the Municipal Solid Waste (MSW) in 2006 in Europe (http://www.waste-management-world.com/index/display/article-display/304406/articles/waste-management-world/volume-8/issue-4/features/msw-management-in-europe.html. Accessed June 2011). Currently, landfilling is the dominant method of packaging waste disposal, followed by recycling, incineration, and composting. Even though recovery methods such as reuse, recycling and/or composting are encouraged as a way of reducing packaging waste disposal, there is still much work to do to substantially reduce the quantity of plastic present in MSW (Jamshidian et al., 2010). Nevertheless, commercial biopolymers show some limitations in terms of performance (thermal resistance, poor barrier, and brittleness) as well as relatively high prices, which are limiting their current applications, requiring modification by addition of other components to improve their performance in food packaging applications.

One of the most studied issues when talking about a package, not just looking for a simple container but also to improve the foodstuff characteristics, are listed below:

1. Allowing a slow but controlled respiration (reduced O_2_ absorption) of foodstuff through the packaging material;
2. Allowing a selective barrier to gases (in particular CO_2_) and water vapor;
3. Creating a modified atmosphere with respect to the internal gas composition, regulating the ripening process, and leading to food shelf-life extension;
4. Lowering the lipids migration, avoiding the packaging modification;
5. Maintaining the polymers structural integrity to improve mechanical handling and processing;
6. Serving as a vehicle to incorporate food additives (flavor agents, colorants, antioxidants, antimicrobial agents);
7. Preventing (or reducing) microbial spoilage during extended storage and distribution (Tharanatha, 2003).

Food industry, and particularly all these companies producing materials for packaging, is subject to pressure from different stakeholders in the production and distribution chain. Producers, retailers and customers have different priorities, and packaging is not always perceived as adding value to the product. But food industry should invest time and energy in defending of packaging with reduced waste across the whole supply chain (Barlow and Morgan, 2013). This is the main reason to think on the use of agricultural and food processing wastes in the packaging industry, while many research institutions have focused their strategy consisting of two issues:

- Reduction in environmental pollution by the packaging industry
- Recovery of biopolymers to use them in the preparation of biodegradable materials (Prochoñ and Przepiórkowska, 2013).

It is well known that bio-based and biodegradable polymers can be classified in four categories depending on the synthetic route followed to get them (Vieira et al., 2011).

1. Polymers obtained from biomass, in particular from agro-resources, such as polysaccharides, e.g., starches (wheat, potatoes, maize) ligno-cellulosic products (wood, straws) and others (pectins, chitosan/chitin, gums) protein and lipids, e.g., animals (casein, whey, collagen/gelatin), and plants (zein, soya and gluten).
2. Polymers obtained by microbial production, e.g., poly(hydroxyalkanoates) (PHAs) such as poly(hydroxybutyrate) (PHB) and poly(hydroxybutyrate co-hydroxyvalerate (PHBV);
3. Polymers chemically synthesized using monomers obtained from agro-resources, e.g., poly(lactic acid) (PLA);
4. Polymers whose monomers are obtained by chemical synthesis from fossil resources, e.g., poly(ε-caprolactone) (PCL), poly(esteramides) (PEA), aliphatic co-polyesters (e.g., PBSA) and aromatic co-polyesters (e.g., PBAT).

The most widely studied thermoplastic biopolymers are starch, PLA and PHAs. Among these, starch and PLA biopolymers are the most interesting for food packaging since they have become commercially available, have an interesting balance of properties, and are produced on an industrial scale. For instance, PLA shows excellent transparency and relatively good water resistance (Park et al., 2002; Chen et al., 2003; Tsuji and Yamada, 2003). Its high stiffness is usually reduced by the addition of plasticizers (Martin and Averous, 2001; Ljungberg and Wesslén, 2002, 2003), but these additives also lead to a decrease in oxygen barrier and thermal resistance (Martino et al., 2009; Courgneau et al., 2011). It is therefore of great industrial interest to enhance the barrier properties of PLA while maintaining its inherently good properties such as transparency and biodegradability. This is one of the main challenges at present in the development of biocomposites for food packaging applications.

There are other biomaterials with a high potential in food packaging and which can be directly extracted from biomass, such as gluten, zein, prolamine obtained from corn and chitosan, which is typically obtained from crustaceous chitin. These materials generally have excellent oxygen barrier under dry conditions. However, their main drawbacks are their inherent high rigidity, difficulties in processing using conventional equipment and strong water sensitivity arising from their hydrophilic character, which leads to plasticization affecting mechanical and barrier properties (Anderson and Lamsa, 2011; Rogovina et al., 2011). Nevertheless, chitosan and zein biopolymers exhibit two very interesting characteristics. Chitosan has antimicrobial properties (Aider, 2010; Campos et al., 2011) and the unusual water resistance of zein makes this biopolymer potentially useful for multilayer food packaging applications (Wang and Padua, 2004).

All biopolymers with commercial interest can show excellent gas barrier properties in their optimum formulations, although their barrier performance is dramatically reduced in the presence of moisture and/or other plasticizers, necessary to get materials adequate for processing. Other biopolymers like PHAs, show very high water barrier properties. So, in principle, the use of multilayer systems where an inner layer of plasticized chitosan could be sandwiched between PHA layers could be an interesting possibility. However, these materials normally suffer from relatively high production costs, meaning that competition with conventional thermoplastics is still problematic. Therefore, the addition of natural additives and/or agricultural waste by-products discarded from food processing operations is an innovative trend in polymer science with clear practical interest.

In this review we summarize these new approaches and the main recent developments in this area are presented.

## Use of natural extracts in biodegradable polymers

New developments in food packaging materials have been recently implemented by the increase in consumers demand for minimally processed foods and stricter requirements regarding consumer health and safety. In fact, new technologies are being investigated in this research line, such as modified atmosphere packaging and active packaging technologies (Suppakul et al., 2003).

Different natural compounds have been proposed for incorporation to the polymer matrices to improve the packaging's functionalities as well as the food quality and safety. Their action is essential in reducing or even eliminating some of the main food spoilage causes, such as rancidity, color loss/change, nutrient losses, dehydration, microbial proliferation, senescence, gas build-up, and off-odors (López-Gómez et al., 2009).

**Figure 1:**
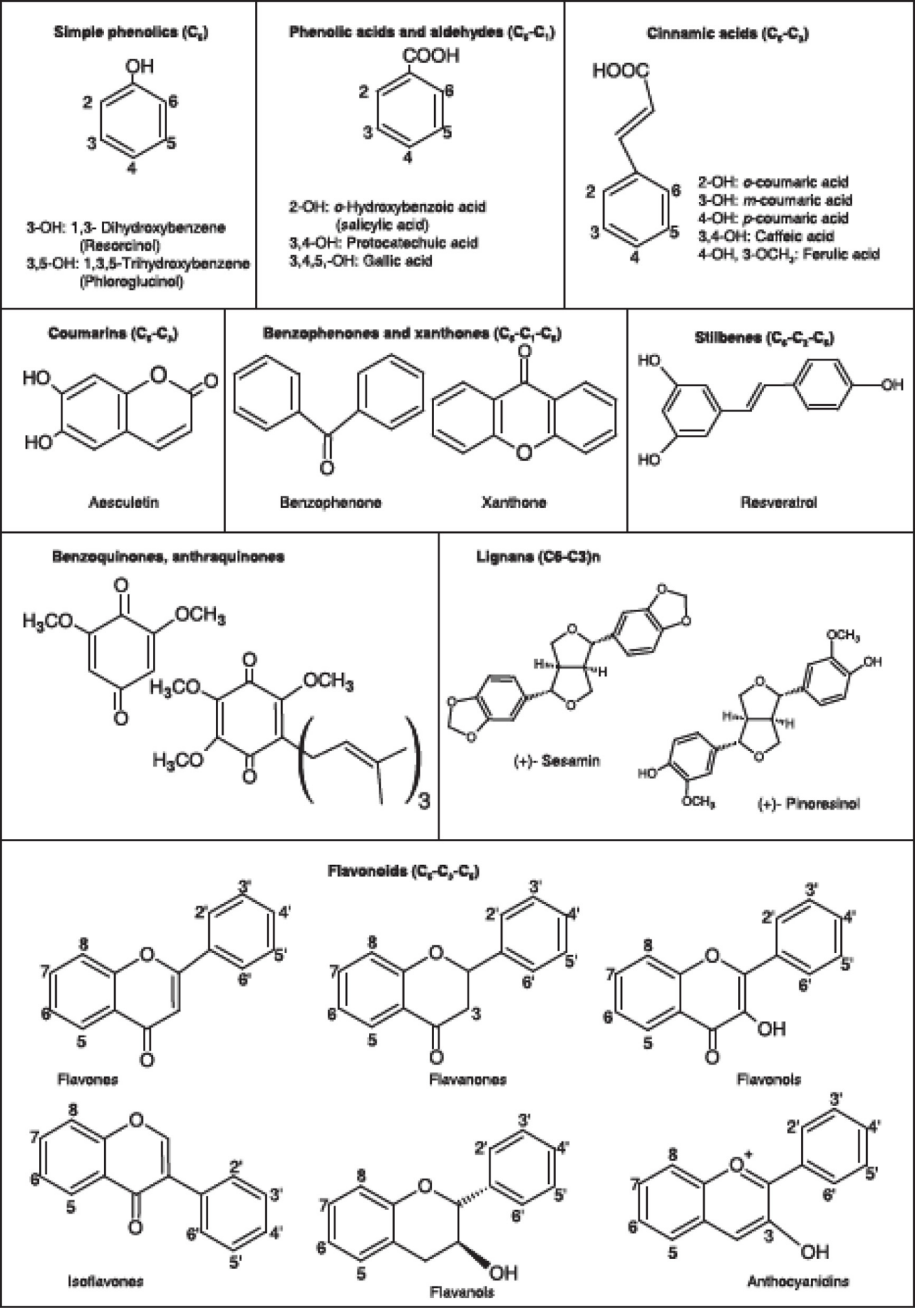
**Phenol derivatives (C6) commonly found in industrial wastewaters or fractions isolated from vegetal sources.** Representative examples are indicated under the general chemical structures (Soto et al., 2011) (with permission).

The demand for the use of natural additives in polymer formulations has produced in recent years a clear increase in the number of studies based on natural extracts which come from plants, essential oils or agricultural waste products (Coma, 2008; Gutiérrez et al., 2009a; Kuorwel et al., 2011). A large number of studies have described the broad antimicrobial spectrum against different pathogenic and spoilage microorganisms, including Gram-negative and Gram-positive bacteria and molds. For instance, extracts of blueberry obtained from four cultivars have shown an antimicrobial effect on the growth of *Listeria monocytogenes* and *Salmonella Enteritidis* (Shen et al., 2014). Grape seed extracts can be used as additives for its well documented anti-inflammatory and antimicrobial properties against major food borne pathogens like *L. monocytogenes*, *Salmonella Typhimurium*, *Escherichia coli* (*E. coli*), and *Campylobacter Jejun* in preventing pathogen contamination. A green tea extract can inhibit the growth of various strains of *Staphylococcus* and some Gram negative bacteria, such as *E. coli* or *Salmonella* (Perumalla and Hettiarachchy, 2011). A recent study demonstrated the promising antimicrobial effects on food of three extracts from raspberry fruits and pomace, including against *E. coli*, *Salmonella sp*., *L. monocytogenes*, *Enterococcus Faecium* (Caillet et al., 2012). These additives are considered to be safe and have the “Generally Recognized As Safe” (GRAS) status as designated by the American Food and Drug Administration (Kuorwel et al., 2011). A summary of the most relevant additives extracted from natural sources is shown in Figure 1.

Due to these beneficial properties, the use of natural extracts or their original compounds (including low molecular weight phenolic acids, tannins, proanthocyanidins, flavonoids, such as anthocyanins or flavonols) for food packaging applications is growing in the last years (Smith-Palmer et al., 2001; Bañón et al., 2007; Carpenter et al., 2007; Negi, 2012). But, sometimes these chemicals are too volatile to be directly incorporated to foodstuff. For this reason a viable alternative is to incorporate these compounds into packaging materials as natural active additives with the possibility to be released to food (Burt, 2004; Del Nobile et al., 2009; Gutiérrez et al., 2009a). In this sense, the antimicrobial active substances are incorporated into the polymer matrix and then they can be gradually released from the packaging material onto the food surface during the total shelf-life of the packaged foodstuff increasing their effect with the aim to control the minimum inhibitory concentration needed for bacterial growth inhibition (Gutiérrez et al., 2009a).

Different types of biopolymers can be used in the development of antimicrobial active films. For instance, Iturriaga et al. used two different biopolymer matrices (gelatin and methyl cellulose) to develop antimicrobial active edible films with citrus extract. These formulations showed convenient properties (lack of odor, water solubility) and interesting antibacterial activity shown against three bacteria by using the agar diffusion method, measuring the inhibition halo against the tested microorganisms. These results showed that the inhibitory effectiveness of the films against the tested strains was maintained regardless of the biopolymer matrix for at least one month (Iturriaga et al., 2012).

Other edible films as soy protein isolate formulations containing grape seed extracts were developed by Sivarooban et al. who demonstrated that these biocomposites were able to reduce the populations of bacteria related with food (*E. coli O157:H7* and *Salmonella Typhimurium* were reduced by 1.8 and 0.6 log CFU/mL, respectively). The incorporation of these extracts significantly increased the films thickness (from 33.0 to 77.9 μm), puncture strength (from 2.5 to 5.3 N), and tensile strength (8.8–10.7 MPa) in comparison to the pure soy protein isolate film (Sivarooban et al., 2008). Tensile properties could be improved by the addition of the natural extracts due to their intercalation in the polymer matrix chains resulting in higher mechanical resistance. Another example was the addition of grapefruit seed extracts to obtain active films based on barley bran protein and gelatin to pack salmon fish. After 15 storage days results showed a significant decrease in the populations of *E. coli O157:H7* and *L. Monocytogenes* compared to the control neat materials (Song et al., 2012). The combination of mint extracts or pomegranate peel extracts with chitosan and polyvinyl alcohol resulted in an improvement of their tensile strength without significantly affecting their barrier properties and effectiveness against Gram-positive food bacteria (Kanatt et al., 2012).

Synthetic biodegradable polyesters, such as PCL, were also used as polymer matrices to produce active food packaging films with the combination of natural extracts which could come from plants or agricultural waste. For instance, Takala et al. based their study in the development of PCL/Alginate films containing three natural extracts from rosemary and Asian and Italian essential oils. The potential in controlling/inhibiting the growth of food borne pathogens in fresh-cut vegetable was studied. Results showed that these composites might be affected by relative humidity in packaging, with the result of altering the mechanism of release of volatile compounds and consequently their antimicrobial activity and films properties (Takala et al., 2013).

Other natural extracts were tested by the need of increasing the value of some sub-products or by-products coming from agricultural waste or food processing residues. For instance, lemon extracts were used by Del Nobile et al. to develop active materials for packaging based on PLA, PCL, and LDPE. In this case the processing temperature of PCL played an important role, since it was necessary to reduce it drastically to avoid the thermal degradation of the polymer matrix and to reduce the risk of the inactivation of the active compounds or volatilization of the lemon extracts (Del Nobile et al., 2009). Propolis extracts were tested in combination with PLA by Mascheroni et al. with the aim to develop an anti-microbial/antioxidant release system for food packaging (Mascheroni et al., 2010). Olive leafs are other important source of active compounds, and the extract showed high antimicrobial efficiency, caused by their high concentration in polyphenols. For this reason, Erdohan et al. studied the incorporation of olive leaf extracts into a PLA matrix. Antimicrobial tests showed that the increase in the amount of the extract could cause a significant increase in inhibitory zones. Moreover, the water vapor permeability, the water solubility, and the degradation rates were modified with the increase of the extracts concentration (Özge Erdohan et al., 2013).

An alternative to composite materials in formulations for food packaging is the use of multilayer systems based on biodegradable layers in combination with natural extracts. Thus, Takala et al. developed trilayer composite films based on methylcellulose (MC) and PCL. The incorporation of natural extracts, such as rosmarinic acid, showed their potential application to control food pathogens, as well as a clear decrease of barrier properties and no significant modifications on tensile strength (Takala et al., 2013).

The addition of natural additives to polymer matrices for food packaging allows their sustained release (migration) to foodstuff during long periods of time (including storage and distribution operations), extending the shelf-life of the final product by the decrease in food autooxidation or spoilage, affecting both sensory and nutritional qualities of food (Manzanarez-López et al., 2011). Migration is the result of diffusion, dissolution, and equilibrium processes involving the mass transfer of low molecular mass compounds initially present in the package into a food sample or food simulant; and it is often described by Fick's second law (Manzanarez-López et al., 2011). The determination of migration of additives in food samples is, in most cases, time consuming and expensive. Thus, migration studies are usually performed using food simulants and conditions specified in European food packaging regulations (Kuorwel et al., 2013).

## Revalorization of agricultural residues as reinforcement in biopolymers

The fabrication of green composites formed by biopolymers and natural fibers has attracted major interest in polymers and composites research. The public concern about the environment, climate change and global warming while limited fossil fuel resources are available, has been important driver for governments, companies and scientists to find alternatives to crude oil in plastics production. Bio-composites obtained from biopolymers and reinforced with natural fibers may offer important contributions by reducing the dependence on fossil fuels and the related environmental impacts. In particular, the use and valorization of agricultural waste is currently a trending topic in research and a raising number of results in this area are being reported.

**Figure 2:**
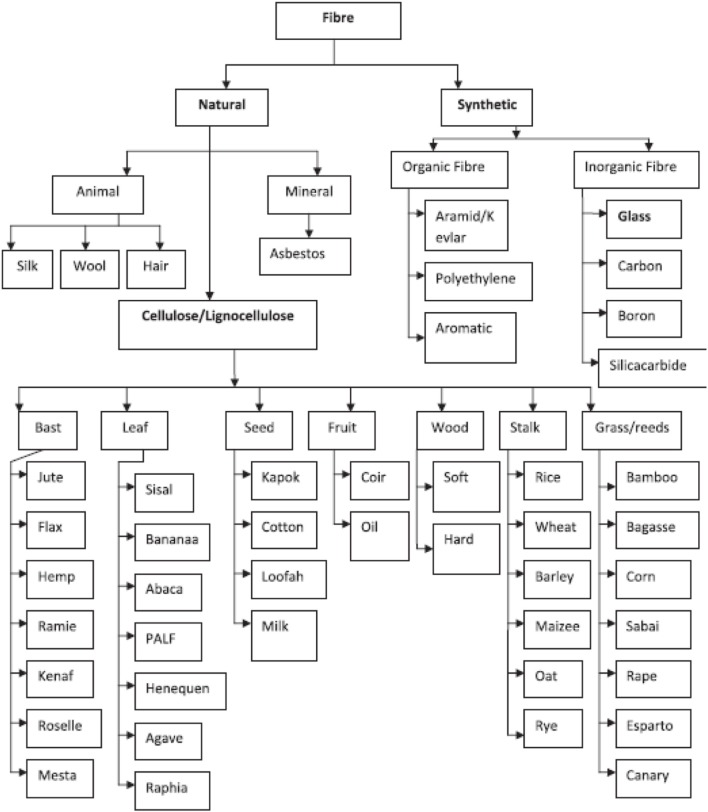
**Fibers classification (Majeed et al., 2013) (with permission)**.

Natural fibers are materials extracted from substances in Nature that can be classified into three main categories: vegetable (plant fibers), animal and mineral fibers (Figure 2). Vegetable or plant fibers are classified into three main subdivisions: leaf fibers (e.g., oil palm, banana, sisal, pineapple, abaca leaf…); bast fibers (e.g., kenaf, jute, and flax); seed fibers (e.g., cotton, rice husk, kapok, and coir). Fibers can be considered as naturally occurring composites constituted mainly of holocellulose (cellulose, hemicellulose) and lignin, with minor contents of sugars, starch, proteins, extractives, and ash (Hamza et al., 2013). The unidirectional cellulose microfibrils constitute the reinforcing elements in the matrix blend of hemicellulose and lignin (Faruk et al., 2012). Several advantages of incorporating natural fibers into biopolymer matrices are interesting to note, since due to their low density and cost, availability, recyclability, environmental friendliness, total degradation in soil without emission of toxic compounds in composting condition, and good mechanical properties, have permitted scientists worldwide to show interest in exploiting the full potentials of plant fibers.

In recent years, researchers have been involved in valorizing agricultural residues and lignocellulosic fibers as polymer reinforcements in substitution of those obtained from glass and carbon. For example, the outer covering of the hull of the soybean seed is a by-product generated during the soybean oil processing and this residue was demonstrated as a good reinforcement to obtain engineer green composites in poly(3-hydroxybutyrate-co-valerate) and polylactide blends (60:40 wt%) (Nanda et al., 2013). This study showed that the addition of 30% of soybean hull induced an important increase in tensile modulus of these composites that may be attributed to the high stiffness of fibers, indicating the efficient transfer of stress from the matrix to the fiber phase; hence an efficient stress transformation is expected in these composites. The potential use of incorporating lignocellulosic agricultural residues, such as soy stalk, corn stalk, wheat straw, and perennial grasses, like switchgrass and miscanthus, as reinforcement into a biodegradable polymer matrices comprising a poly(3-hydroxybutyrate-co-3-hydroxyvalerate) and poly(butylene adipate-coterephthalate) blend has been recently investigated (Nagarajan et al., 2013). The incorporation of miscanthus, with high cellulose amounts which allowed better interactions with the matrix, resulted in increased tensile strengths of composites. The flexural strength of these composites with lignocellulosic fibers obtained from agricultural wastes improved significantly, with the exception of the switchgrass-based composite. Finally, the moduli of these green composites increased drastically with no significant differences between fiber types. However, the elongation at break of bio-composites was found to be lower than 5% regardless of the bio-fiber type and due to the restriction of the molecular mobility of the polymer chains in the composite. In addition, the impact strength was reduced by two mechanisms: (1) the drastic reduction in elongation at break, reducing the area under the stress-strain curve, and (2) the stress concentration at areas of poor adhesion around fiber ends and fiber-fiber contacts.

A preview review (Wahit et al., 2012) highlighted developments in the production of PLA and PCL eco-friendly composites with enhanced mechanical properties and biodegradation with the incorporation of natural fibers such as hemp, wood, ramie, kenaf, rice straw, abaca, jute, bamboo, rice husk, oil palm, and flax. In this sense, a comparative study of the mechanical properties of PLA/kenaf and PLA/rice husk composites showed the remarkable increase in flexural modulus. For the PCL/flax fiber composite, tensile strength, and flexural strength increased by 54 and 44%, respectively. PCL/rice husk composite decreased in melting temperature and crystallinity at high fiber contents. Moreover, the high-level degradation for all PCL/rice husk composites after 57 days of incubation was achieved since the addition of rice husk facilitated the access of water to the PCL matrix as well as the controlled absorption and stabilization of PCL depolymerase, resulting in a clear increase of the PCL hydrolytic degradation. The morphology, crystallization behavior, thermal, mechanical and barrier properties and biodegradation in soil of fully biodegradable bio-composites with 5 and 15 wt% of cotton, cellulose obtained from cotton and hydrolyzed cellulose into a PCL matrix was also investigated (Gutiérrez et al., 2009b). It was concluded that the incorporation of lignocellulosic fillers drove to a decrease on the tensile strength and elongation at break, as well as an increase on the elastic modulus. The cellulose obtained from cotton residues, due to their lower hidrophilicity, showed the highest compatibility with PCL, while strongly hydrophobic reinforcement fillers resulted in increased mechanical properties (Faruk et al., 2012). The best performance was obtained with the addition of 15 wt% of cellulose obtained from cotton residues, which showed the highest toughness and did not change the gas barrier properties of the neat matrix. The biodegradation processes were accelerated by the presence of these fillers but the effect was not severe. Therefore, these composites could be useful for packaging applications. Similar behavior was reported in valorising different agricultural residues, such as walnut shells (Pirayesh et al., 2012), almond shells (Pirayesh et al., 2013), orange tree pruning fibers (Reixach et al., 2013), bamboo (Abdul Khalil et al., 2012), rice-husk fiber, bagasse fiber and waste fish in polypropylene (Nourbakhsh et al., 2014), jute, flax and wood, among others, as reinforcement in polymeric composite materials for different industrial applications.

Composites reinforced with agricultural residues have been object of raising interests in the development of active packaging systems. For example, ethylene-scavenging packaging materials have been subject to recent studies to be applied in the fresh fruit and vegetables industry. Commercial ethylene scavengers are normally based on potassium permanganate (KMnO_4_) but a clear limitation on their application with regard to food is that KMnO_4_ is not allowed to come into contact with foodstuff because of its toxicity and color. In order to overcome this problem, composites with rice straw incorporating activated carbon were proposed and their effect on the physical and mechanical properties in the resulting composites was reported (Sothornvit and Sampoompuang, 2012). Results showed that the incorporation of 30% activated carbon to rice straw paper produced the maximum level of ethylene scavenging (77%) in environmentally-friendly way owing to its reusability and protecting against mechanical damage.

## Legislative issues

Due to interactions with food and/or the environment, active packaging represents a new challenge to progress in advanced materials for research. The intentional migration of active elements through the packaging materials and to food would fall under the Framework Regulation 1935/2004 for active packaging materials (European Commission, 2004). This Regulation already contained general provisions on the safety of active packaging and set the framework for the EFSA's safety evaluation process (Dainelli et al., 2008). But some articles, such as active and intelligent materials, fall within more specific regulations. In this sense, the Commission Regulation (EC) No 450/2009 (European Commission, 2009) was related to active and intelligent packaging and it pointed out that the active element needs to be identified and the active material has to be accompanied by information on the permitted uses. The maximum quantity of substances released by the active component should be specified (Llorens et al., 2012).

It should be highlighted that the amount of active compounds released from packaging materials could exceed the overall migration requirements indicated in the EU or national legislations. The transfer of these active substances to food should not be included in the calculation of the overall migration limit (OML). However, passive parts should be covered by the specific legislation applicable to those materials, such as the EU Regulation 10/2011. Only the active components should be subjected to safety assessment by the EFSA (European Food Safety Authority) before they are authorized for their use, and a list of substances or group/combination of substances to be used in active and intelligent materials should be drawn up following the risk assessment of these substances by EFSA.

Some Guidance to the Commission Regulation (EC) No 450/2009 was provided by the European Commission to deal with questions concerning the interpretation and implementation of certain aspects in the referred legislation (European Commission, 2011). This document contains some definitions and examples; clarification on packaging types and components that fall or not fall under the definition of active materials; legal aspects in related to the authorization of active substances or components; and questions and answers related to the risk assessment and authorization procedure by EFSA. Factors considered by the authority when making safety assessments include, for example, toxicological properties and the extent to which the original or their breakdown products could transfer into food.

EFSA guidelines are only applicable to substances in direct contact with food or the environment surrounding the food (headspace). These guidelines are also applied to materials which are separated from food by a functional barrier (i.e., a barrier consisting of one or more layers of food-contact materials which ensures that the finished material or article complies with Article 3 of Regulation 1935/2004/EC and with Regulation 450/2009/EC). Substances behind such a barrier do not need a safety evaluation and are also outside the scope of these Regulations. Therefore, behind a functional barrier non-authorized substances may be used if their migration does not exceed 0.01 mg kg^−1^ food and they are not carcinogenic, mutagenic, or toxic to reproduction.

Considering regulatory requirements for new active packaging technologies in United States, materials used in food-contact applications are subject to pre-market regulatory clearance by the US Food and Drug Administration if they are deemed “food additives.” Some organic acids, bacteriocins and volatile compounds derived from plants have FDA approval as additives for certain foods (see Table 1) (Suppakul et al., 2003).

**Table 1:** **List of some permitted food additives that could be used as active agents in packaging materials (Suppakul et al., 2003) (with permission)**.

**Additive**	**Code assigned by legislative authority**		
	**Australia/New Zealand^1^**	**Europe^2^**	**USA^3^**
Acetic acid	260	E260	GRAS
Benzoic acid	210	E210	GRAS
Butylated hydroxyanisole (BHA)	320	E320	GRAS
Butylated hydroxytoluene (BHT)	321	E321	GRAS
Carvarcol			FA
Citral			GRAS
Citric acid	330	E330	GRAS
*p*-Cresol			FA
EDTA			FA
Estragloe (methyl chavicol)			GRAS
Ethanol		E1510	GRAS
Ethyl paraben		E214	GRAS
Eugenol			GRAS
Geraniol			GRAS
Glucose oxidase	1102		GRAS
Hexamethylenetetramine (HMT)		E239	
Konjac glucomannan		E425	GRAS
Lactic acid	270	E270	GRAS
Lauric acid			FA
Linalool			GRAS
Lysozyme	1105	E1105	GRAS
Mallic acid	296	E296	GRAS
Methyl paraben	218	E218	
Natamycin	235	E235	FA
Nisin	234	E234	GRAS
Phosphoric acid	338	E338	GRAS
Polyphosphate		E452	GRAS
Potassium sorbate	202	E202	GRAS
Propionic acid	280	E280	GRAS
Propyl paraben	216	E216	GRAS
Sodium benzoate	211	E211	GRAS
Sorbic acid	200	E200	GRAS
Succinic acid		E363	GRAS
Sulfur dioxide	220	E220	GRAS
Tartaric acid	334	E334	GRAS
Teritiary butylhydroquinone (TBHQ)	319		FA
α-Terpineol			FA
Thymol			FA

Source: CFR (1988); Davidson and Branen (1993); Maga and Tu (1995); Luck and Jager (1997); Saltmarsh (2000); Taubert (2000).

1 Assignment of a number signifies that additive is approved by the Australian and New Zealand Food Authority (ANZFA) and The Australian New Zealand Food Standards Council (ANZFSC) as being safe for food use.

2 Assignment of an “E” number signifies that additive has been approved by the European Communities (EC), Scientific Committee on Food (SCF).

3 Classification in accordance with Food and Drug Administration (FDA) Title 21 of the code of Federal Regulations (21 CFR) wherein substances intended for use in the manufacture of foodstuffs for human consumption are classified into 3 categories: food activities (FA), prior-sanctioned food ingredients and substances generally recognized as safe (GRAS).

**Figure 3:**
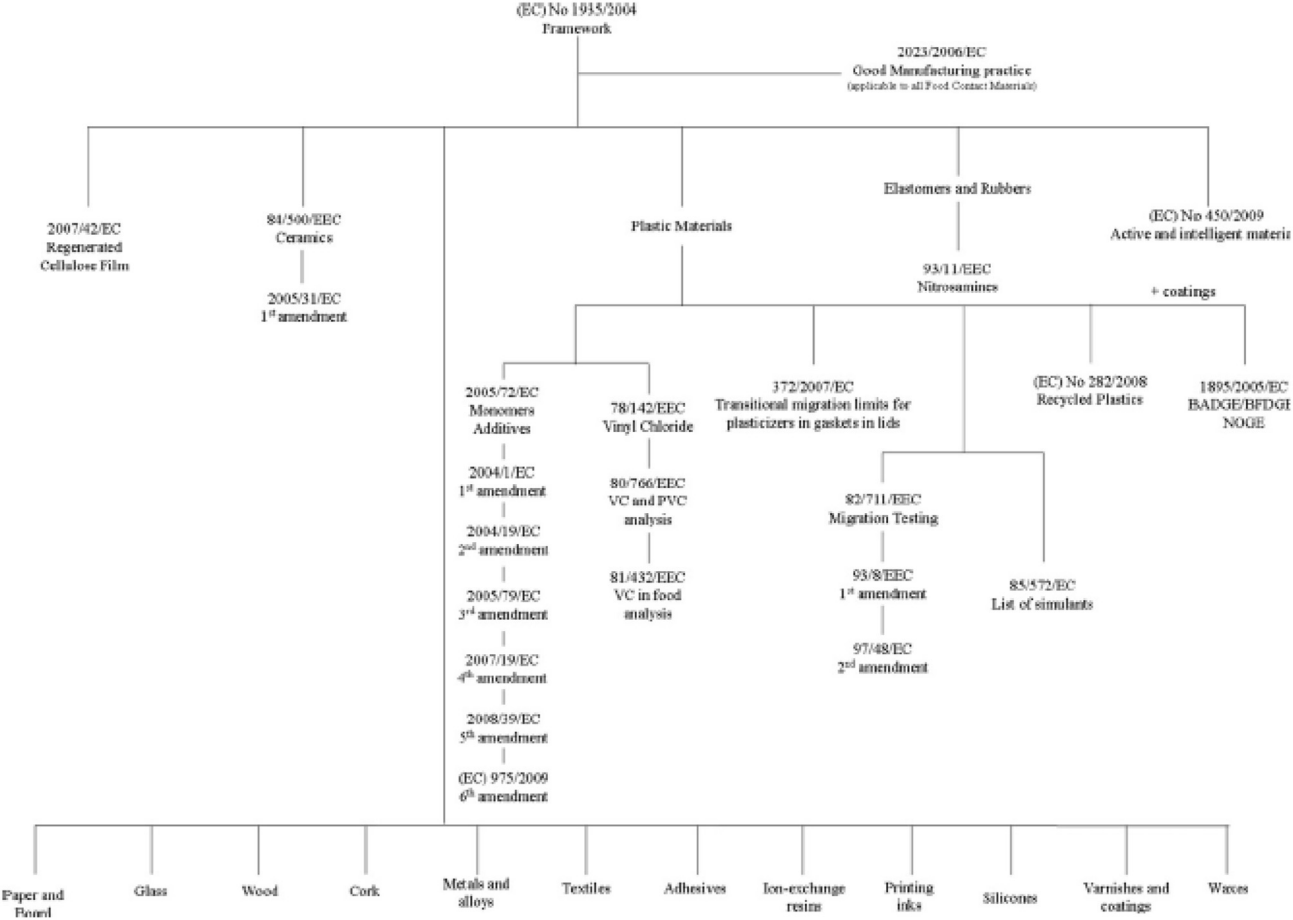
**EU food-contact materials legislation (Restuccia et al., 2010) (with permission)**.

In summary, Regulations 1935/2004/EC and 450/2009/EC poses new basis for the general requirements and specific safety and marketing issues related to active packaging materials. In this sense, it should be considered that complexity of new developed systems introduce many variables into risk assessment; introducing new migration products and leading to different interactions between active agents and different packaging materials. The development and validation of migration tests to reliably detect and measure new migration products could represent a serious challenge, as well as the risk assessment for nanomaterials (Restuccia et al., 2010). The main legislation associated with active materials in shown in Figure 3.

### Conflict of interest statement

The authors declare that the research was conducted in the absence of any commercial or financial relationships that could be construed as a potential conflict of interest.
